# The Growth Supporting Role of ZDHHC11 Is Linked to the MEF2B–BCL6 Regulatory Circuit in Burkitt Lymphoma

**DOI:** 10.1002/ijc.70506

**Published:** 2026-04-16

**Authors:** Lotteke J. Y. M. Ziel‐Swier, Karolina Rassek, Yichen Liu, Annika Seitz, Jasper Koerts, Debora de Jong, Bea Rutgers, Anastasiia Kompaniiets, Julia Przybył, Martine E. D. Chamuleau, Anke van den Berg, Agnieszka Dzikiewicz‐Krawczyk, Joost Kluiver

**Affiliations:** ^1^ Department of Pathology and Medical Biology University of Groningen, University Medical Center Groningen Groningen the Netherlands; ^2^ Institute of Human Genetics, Polish Academy of Sciences Poznan Poland; ^3^ Department of Hematology, Cancer Center Amsterdam Amsterdam UMC Amsterdam the Netherlands

**Keywords:** BCL6, Burkitt lymphoma, MEF2B, ZDHHC11

## Abstract

We previously showed that *ZDHHC11* promotes cell growth in Burkitt lymphoma (BL). To explore the underlying mechanism, we performed a genome‐wide gene expression analysis upon *ZDHHC11* knockdown. We identified MEF2B, a transcription factor critical for germinal center formation, as a downstream target and validated repression of MEF2B at the RNA and protein level upon *ZDHHC11* knockdown in BL cell lines. The relevance of MEF2B was shown upon its knockdown, which strongly inhibited growth of BL cells. Since MEF2B is a known regulator of BCL6 in normal GC B‐cells, diffuse large B‐cell lymphoma, and follicular lymphoma, we subsequently focused on the effect of MEF2B knockdown on BCL6. We observed a strong decrease in BCL6 at the RNA and protein level in BL and showed a strong correlation between MEF2B and BCL6 transcript levels in a panel of B‐cell lymphoma cell lines, primary BL samples, and normal B‐cell subsets. Knockdown of BCL6 also strongly inhibited growth of BL cell lines, whereas BCL6 overexpression partially rescued the growth‐inhibitory effect of MEF2B knockdown. Together, our data indicate that ZDHHC11 promotes BL cell growth at least in part by stimulating expression of MEF2B, which promoted BL cell survival through both BCL6‐dependent and independent pathways. Our work highlighted the importance of the MEF2B‐BCL6 axis, which strongly supports BL growth and identified ZDHHC11 as a novel regulator of this axis.

## Abstract (highlights)

What's new?

MEF2B and BCL6 have established functions in germinal center B‐cell biology and lymphomagenesis. The mechanism of MEF2B deregulation and its significance for cell survival in Burkitt lymphoma however remain unclear. This study highlights MEF2B as a crucial transcription factor in Burkitt lymphoma and suggests ZDHHC11 as an upstream regulator of MEF2B. While pointing to the existence of other target genes, the results confirm BCL6 as a key downstream target of MEF2B with a growth supporting role. The study highlights the importance of the MEF2B‐BCL6 axis in Burkitt lymphoma and identifies ZDHHC11 as a novel regulator of this axis.

## Graphical abstract

MEF2B and BCL6 have established functions in germinal center B‐cell biology and lymphomagenesis. The mechanism of MEF2B deregulation and its significance for cell survival in Burkitt lymphoma however remain unclear. This study highlights MEF2B as a crucial transcription factor in Burkitt lymphoma and suggests ZDHHC11 as an upstream regulator of MEF2B. While pointing to the existence of other target genes, the results confirm BCL6 as a key downstream target of MEF2B with a growth supporting role. The study highlights the importance of the MEF2B‐BCL6 axis in Burkitt lymphoma as well and identifies ZDHHC11 as a novel regulator of this axis.

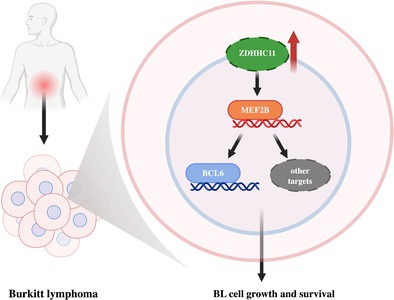

**Abbreviations**
BCL6: BCL6 Transcription Repressor
BL: Burkitt lymphoma
CRISPR: clustered regularly interspaced short palindromic repeats
DLBCL: diffuse large B‐cell lymphoma
FL: follicular lymphoma
GC: germinal center
GFP: green fluorescent protein
KO: knockout
MEF2B: Myocyte Enhancer Factor 2B
MYC: MYC Proto‐Oncogene, BHLH Transcription Factor
NHL: non‐Hodgkin lymphoma
RT‐qPCR: reverse transcription‐quantitative polymerase chain reaction
sgRNA: single guide RNA
shRNA: short hairpin RNA
ZDHHC11: ZDHHC palmitoyltransferase 11

## 1 Introduction

B‐cell non‐Hodgkin lymphomas (B‐NHLs) are a highly heterogeneous group of mature B‐cell malignancies. Among various subtypes of B‐NHLs, diffuse large B‐cell lymphoma (DLBCL) and follicular lymphoma (FL) constitute the two most common forms, while Burkitt lymphoma (BL) is the most aggressive [1]. BL originates from germinal center (GC) B cells and occurs most frequently in children. The most characteristic feature of BL is the translocation of the *MYC* gene locus to one of the immunoglobulin gene loci, which results in high expression of the oncogenic transcription factor MYC [2, 3]. Although B‐NHLs represent clinically and phenotypically distinct malignancies with unique molecular pathogenesis and characteristic hallmark translocations, several recurrently altered genes have been identified across multiple subtypes. These include the above‐mentioned *MYC* (common for BL and DLBCL), *BCL2* (shared by FL and DLBCL), *BCL6, KMT2D*, *CREBBP*, and *EZH2* (observed in FL and DLBCL) [2, 4, 5, 6, 7, 8, 9, 10, 11].

The myocyte enhancer‐binding factor 2B (MEF2B) is a member of the BCL6 transcriptional complex and functions as a transcriptional regulator of germinal center (GC) B‐cell development. It belongs to the MEF family which includes three additional members (MEF2A, MEF2C, and MEF2D) [12]. MEF2 transcription factors contribute to the development of many cell types (e.g., muscle, heart, hematopoietic, and immune cells) by regulating their differentiation, proliferation, apoptosis, migration, morphology, and metabolism [13, 14, 15, 16]. MEF2 proteins have been identified as either oncogenes or tumor suppressors in several types of cancer, reflecting their context‐dependent roles in tumorigenesis [17, 18, 19]. In mature B cells, two major MEF2B isoforms are expressed, i.e., isoform A (isoA) and isoform B (isoB), which differ from amino acid 257 to the C‐terminus [12]. MEF2B regulates expression of genes by binding to active chromatin regions (marked by H3K4me3 and H3K27ac), thereby influencing processes such as DNA replication and repair, cell cycle and apoptosis [20]. In vivo studies have shown that deletion of *Mef2b* in GC B cells impairs GC formation, whereas deletion of all *MEF2* family members completely abolishes it [21]. These data indicate overlapping or compensatory functions between MEF2B and its paralogs in GC development. MEF2B expression has been shown to positively correlate with BCL6 levels in GC B‐cells and DLBCL [12, 22]. MEF2B mutations have been identified in 11% of DLBCL and 12% of FL cases and are likely essential for the regulation of BCL6‐mediated lymphomagenesis partly via the promotion of BCL6 expression and partly through the activation of the ERK1/2 pathway [12, 23]. In BL, MEF2B mutations are typically absent, despite high expression of MEF2B and BCL6. This suggests that alternative mechanisms driving the MEF2B‐BCL6 axis might be activated in BL and highlights an incomplete understanding of this aspect of BL lymphomagenesis.

We previously identified a critical cell growth regulatory role of the novel MYC/miR‐150/MYB/ZDHHC11 network in BL [24]. ZDHHC11 belongs to the ZDHHC enzyme family, a group of 24 palmitoyltransferases that modulate protein stability, localization, and function [25]. The *ZDHHC11* gene encodes three different transcripts including a mRNA (pcZDHHC11), a linear long noncoding RNA (lncZDHHC11), and a circular noncoding RNA (circZDHHC11) [26, 27]. To obtain insight into how ZDHHC11 promotes growth of BL cells, we performed genome‐wide gene expression analysis upon *ZDHHC11* knockdown and identified MEF2B as one of the downregulated genes. Next, we focused on MEF2B and its downstream target BCL6, characterized their relationship, and determined whether the growth‐promoting effects of MEF2B are mediated through BCL6‐dependent or independent mechanisms.

## 2 Materials and Methods

### 2.1 Cell Lines and Sorting of B‐Cell Subsets

The cell lines used are listed in Table 1, including their origin and culturing conditions. Cell lines were either cultured in RPMI‐1640 (Gibco, USA; in case of ST486 from Lonza BioWhittaker, USA) or DMEM (Gibco), supplemented with 10% or 20% fetal bovine serum (Serana, Germany), 100 U/mL of penicillin (Gibco), 100 μg/mL of streptomycin (Gibco), and 2 mM of glutamine (Gibco). The cultures were maintained at 37°C in humidified air atmosphere supplemented with 5% CO_2_. All cell lines were authenticated using short tandem repeat (STR) profiling within the last 3 years. All experiments were performed with mycoplasma‐free cells.

**TABLE 1:** Cell lines and culturing conditions.

Cell line	Type	EBV	Source	Medium	FBS
HEK293T (CVCL_0063)	human embryonic kidney cell	−	ATCC	DMEM	10%
BL‐41 (CVCL_1087)	BL	−	DSMZ	RPMI‐1640	10%
BL‐65 (CVCL_C163)	BL	+	DSMZ	RPMI‐1640	20%
CA46 (CVCL_1101)	BL	−	DSMZ	RPMI‐1640	10%
DG‐75 (CVCL_0244)	BL	−	DSMZ	RPMI‐1640	10%
Jijoye (CVCL_1317)	BL	+	DSMZ	RPMI‐1640	10%
Namalwa (CVCL_0067)	BL	+	DSMZ	RPMI‐1640	10%
Raji (CVCL_0511)	BL	+	DSMZ	RPMI‐1640	10%
Ramos (CVCL_0597)	BL	−	ATCC	RPMI‐1640	10%
ST486 (CVCL_1712)	BL	−	ATCC	RPMI‐1640	20%
NU‐DUL‐1 (CVCL_1877)	DLBCL, ABC	−	DSMZ	RPMI‐1640	20%
OCI‐Ly3 (CVCL_8800)	DLBCL, ABC	−	DSMZ	RPMI‐1640	20%
Ri‐1 (CVCL_1885)	DLBCL, ABC	−	DSMZ	RPMI‐1640	10%
SU‐DHL‐2 (CVCL_9550)	DLBCL, ABC	−	DSMZ	RPMI‐1640	20%
SU‐DHL‐4 (CVCL_0539)	DLBCL, GCB	−	DSMZ	RPMI‐1640	10%
SU‐DHL‐5 (CVCL_1735)	DLBCL, GCB	−	DSMZ	RPMI‐1640	10%
SU‐DHL‐6 (CVCL_2206)	DLBCL, GCB	−	DSMZ	RPMI‐1640	10%
SU‐DHL‐10 (CVCL_1889)	DLBCL, GCB	−	DSMZ	RPMI‐1640	20%
SU‐DHL‐16 (CVCL_1890)	DLBCL, GCB	−	DSMZ	RPMI‐1640	20%
U‐2932 (CVCL_1896)	DLBCL, ABC	−	DSMZ	RPMI‐1640	10%
WSU‐DLCL2 (CVCL_1902)	DLBCL, GCB	−	DSMZ	RPMI‐1640	10%

GCB‐cells (defined as CD20^+^IgD^−^CD38^+^) were sorted from routinely removed tonsil specimens as described previously [28].

BL tissues (*n* = 13) were randomly selected from a previously conducted phase III HOVON/SAKK trial for newly diagnosed BL patients [29]. Diagnosis of BL was confirmed by central review. Reactive lymph node (*n* = 3) and tonsillar tissues (*n* = 6) were selected from local UMCG pathology files.

### 2.2 DNA Constructs and Viral Transfection

To knock down the expression of all *ZDHHC11* transcripts simultaneously, lentiviral shRNA constructs and nontargeting control shRNA constructs with GFP or RFP reporter described and tested previously were used [24]. For the knockdown of MEF2B or BCL6, sgRNAs targeting MEF2B or BCL6 were cloned into the lentiCRISPRv2GFP vector (Addgene plasmid #82416), which was a gift from David Feldser [30]. Nontargeting sgRNA constructs were used as negative control. The sequences of all shRNAs and sgRNAs are listed in Table S1. Lentiviral particles were generated in HEK293T cells as published previously and were either used directly to infect target cells or stored at −80°C after being harvested [28]. To validate the knockdown efficiency or study the effect of knockdown on the expression of other genes, GFP^+^ or RFP^+^ cells were harvested on day 7–12 after transfection (dependent on the cell line and the phenotype upon knockdown) using a MoFlo XDP or Astrois sorter (Beckman Coulter, USA). To test the shRNA‐induced knockdown efficiencies in BL‐41 and CA46 cell lines, a puromycin selection was performed for 1–1.5 weeks to obtain GFP percentages higher than 95%.

### 2.3 RNA Isolation and Reverse Transcriptase‐Quantitative PCR

Cells were resuspended in Qiazol (Qiagen, USA) and total RNA was isolated using the miRNeasy mini or micro kit (Qiagen) in combination with Phase Lock Gel Heavy tubes (5Prime, Germany) according to the manufacturer's protocol. Synthesis and amplification of cDNA were performed using 500 ng of RNA, random primers, dNTP mix, and the Superscript II Reverse Transcriptase Kit (Life Technologies, The Netherlands) according to the manufacturer's protocol. For quantification of the RNA transcripts, SYBR Green (Applied Biosystems, USA) RT‐qPCR was performed with the primers listed in Table S1, taking 1 ng of cDNA as input in 10 μL reactions. For quantification of the circular *ZDHHC11* transcript, 10 ng of cDNA was used. *TBP* was used for normalization of the expression levels. For quantification of miR‐150 levels, multiplex miRNA‐specific cDNA synthesis and RT‐qPCR were performed using the TaqMan MicroRNA Reverse Transcription Kit and TaqMan MicroRNA Assays (Applied Biosciences) according to the manufacturer's protocol, using *RNU48* for normalization (MicroRNA Assay IDs 000473 (miR‐150) and 001006 (RNU48)).

### 2.4 GFP Competition Assay

To determine the effect on cell growth, lentiviral infections were performed aiming at infection percentages of 15%–50%. The percentages of GFP^+^ or RFP^+^ cells were measured on a BD FACSCalibur flow cytometer or BD Accuri C6 Plus Cell Analyzer (BD Biosciences, USA) at day 4 post‐transfection and monitored tri‐weekly for 3 weeks. Data were analyzed using the FlowJo software (version 10; Treestar, USA). The percentage of GFP^+^ or RFP^+^ cells on day 4 post‐transfection was set to 100%, and the fold difference relative to this starting point was calculated for each time point.

### 2.5 Microarray

Total RNA of ST486 cells transfected with shRNAs targeting all *ZDHHC11* transcripts simultaneously or control shRNAs was isolated as described above and analyzed on an Agilent SurePrint G3 Human GE 8 × 60 microarray (Agilent ID 72363; Agilent Technologies, USA). Labeling and hybridization were performed with 300 ng of total RNA using the LowInput QuickAmp Labeling kit and Cyanine 3 CTP Dye pack (Agilent Technologies) according to the manufacturer's protocol. The data are deposited in the Gene Expression Omnibus (GSE218286) and analyzed using the GeneSpring GX software (version 12.5, Agilent Technologies).

### 2.6 Western Blot

Whole cell protein lysates were prepared using cell lysis buffer (Cell Signaling, USA) supplemented with 1 mM of PMSF or RIPA buffer (Sigma‐Aldrich, St Louis, USA). The lysate was collected by centrifugation following an incubation on ice for 45 min. The total protein concentration was determined using the Pierce BCA Protein Assay Kit (ThermoFisher Scientific, Waltham, MA, USA) according to the manufacturer's protocol. SDS‐PAGE was performed using 8% or 10% polyacrylamide gels with 2,2,2‐Trichloroethanol (TCE) for stain‐free total protein quantification and subsequently, the proteins were transferred onto nitrocellulose membranes. Membranes were blocked in 5% milk in TBST, followed by incubation with antibodies diluted in 5% milk in TBST. Subsequently, incubation with a secondary and tertiary antibody was performed for each membrane. The antibodies used are listed in Table S2. Membranes were incubated with SuperSignal West Pico Chemiluminescent Substrate (ThermoFisher Scientific). The proteins of interest were visualized using a ChemiDoc MP scanner and quantified using the Image Lab 6.0 software (BioRad). Protein levels were normalized to the total amount of protein loaded per lane.

### 2.7 BCL6 Overexpression and Phenotype Rescue Experiment

Lentiviral vector with BCL6 OE under the control of doxycycline‐inducible promoter was purchased from Vector Builder. Cells were selected with blasticidin for 7 days and banked for further experiments. BCL6 overexpression was induced with 1 μg/mL doxycycline for 48 h. For the phenotype rescue experiment, ST486‐BCL6, BL‐41‐BCL6, and CA46‐BCL6 cells were transduced with sgRNAs targeting MEF2B or control sgRNAs. The percentage of GFP^+^ cells was first measured at day 4 post‐transduction, and from this point onward, cells were cultured in parallel with or without doxycycline and measured by FACS tri‐weekly until day 22 post‐transduction. The percentage of GFP^+^ cells on day 4 (ST486) or day 6 (BL‐41 and CA46) post‐transduction was set to 100%, and the fold difference relative to this starting point was calculated for each time point. Mixed model analyses were performed to determine significant differences.

### 2.8 Statistical Analysis

Microarray data were analyzed using the GeneSpring GX software (version 12.5, Agilent Technologies). Quantile normalization of the data was performed, and probes which were not detected in all samples were filtered out. Moderated *T*‐test without multiple testing correction was used to identify probes significantly up‐ or downregulated upon knockdown of all three ZDHHC11 transcripts (*p* < 0.05, FC ≥ 2). For GFP competition assays, mixed model analyses were performed using SPSS (IBM, USA) to determine significant differences between cells with decreased ZDHHC11, MEF2B, or BCL6 levels and cells transfected with control constructs on day 22 post‐transfection [24]. R‐squared Pearson correlation coefficient was calculated using GraphPad Prism to determine the correlation between transcript levels; two‐tailed *p* values are given.

## 3 Results

### 3.1 Knockdown of ZDHHC11 Repressed MEF2B Levels

**FIGURE 1:**
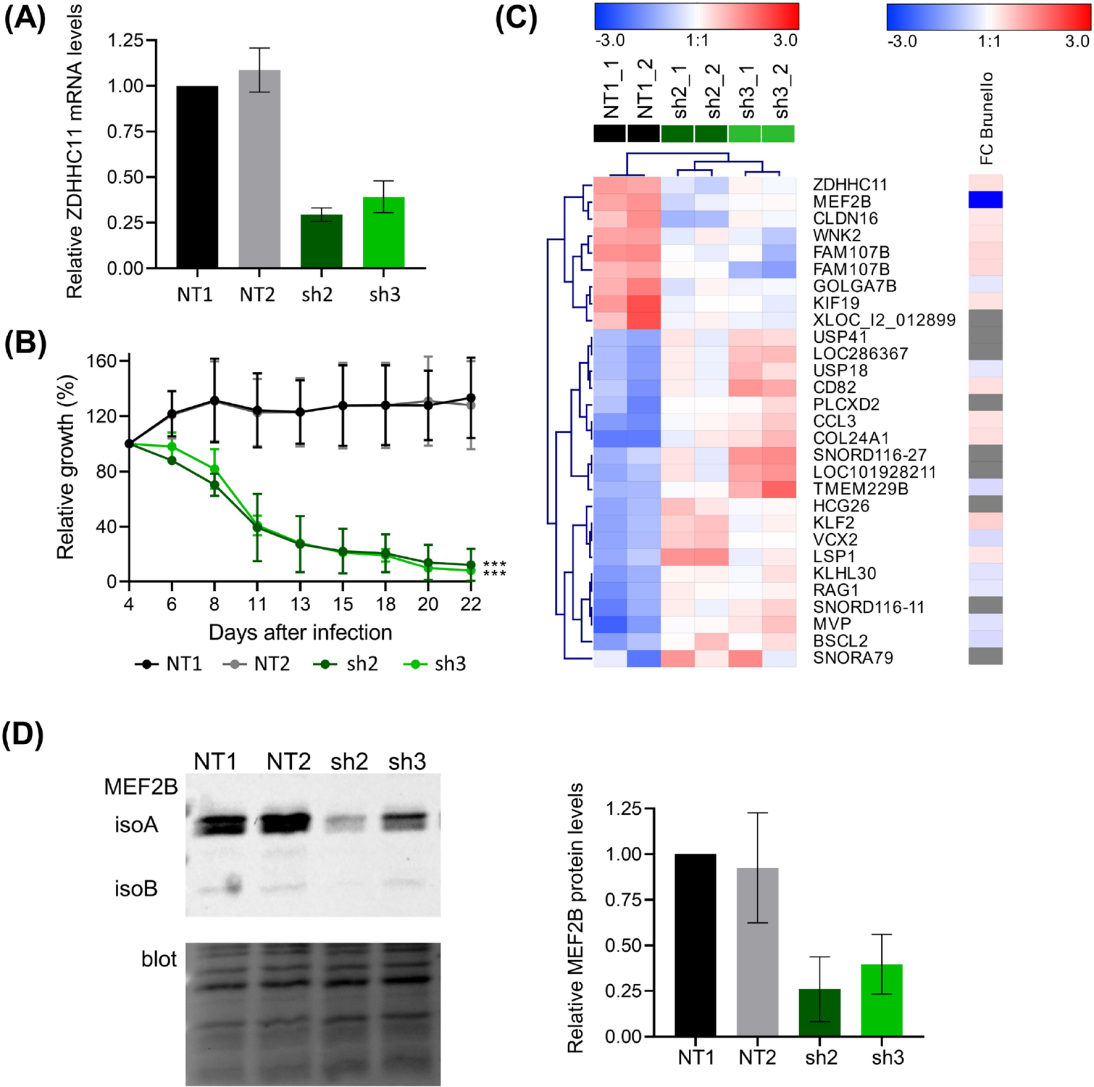
Knockdown of all ZDHHC11 transcripts represses MEF2B levels. ST486 cells were transfected with lentiviral vectors carrying control (NT1: Black and NT2: Grey) or shRNA sequences targeting all three ZDHHC11 transcripts (sh2: Dark green and sh3: Light green). (A) Relative expression of all ZDHHC11 transcripts upon knockdown of the ZDHHC11 transcripts was measured by RT‐qPCR. Mean ± SD of three independent experiments is shown and the data were normalized to the NT1 control. (B) Relative growth of ST486 cells upon ZDHHC11 knockdown was measured by following the percentage of GFP+or RFP+cells over 3 weeks post‐transfection, with the percentages normalized to day 4 after transfection. Mean ± SD of three independent experiments is shown. Significance was determined by mixed model analysis; ***p < 0.001. (C) Hierarchical clustering of the 28 genes significantly differentially expressed upon knockdown of ZDHHC11 (moderated *t*‐test *p* < 0.05). The upper eight, among which ZDHHC11, are downregulated, while the bottom 20 are upregulated. For FAM107B, two probes showed differential expression. The fold changes (FC) of a high‐throughput screen with the human CRISPR Brunello knockout library performed in ST486 [31] are depicted using the same color coding, with grey representing no data available. (D) Western blot shows downregulation of MEF2B levels upon ZDHHC11 knockdown. MEF2B is present in two isoforms, isoA and isoB. Quantification of the most prominent isoA was normalized to the total amount of protein loaded per lane, and the data were plotted relative to the NT1 control sample. Mean ± SD of two independent biological experiments is shown, with each sample loaded twice on gel.

To identify potential gene regulatory effects of ZDHHC11, we performed a microarray analysis upon ZDHHC11 knockdown in the BL cell line ST486. This cell line was selected as we previously showed that it had the strongest negative effect on growth upon ZDHHC11 downregulation [24]. Knockdown of all *ZDHHC11* transcripts (ZDHHC11_all) simultaneously, using short hairpin RNAs (shRNAs), resulted in a 60%–70% decrease of ZDHHC11 transcript levels (Figure 1A). Consistent with our previous study, this induced a strong negative effect on cell growth with a decrease of about 90% at day 22 after infection (Figure 1B). Using a moderate *T*‐test without MTC, we identified 20 up‐ and 8 downregulated genes upon knockdown of ZDHHC11, including ZDHHC11 (Figure 1C and Table S3). To confirm the array data, we validated the differential expression pattern for five up‐ and five downregulated genes (Figure S1 A). To select downstream candidates that might contribute to the growth promoting effect of ZDHHC11, we intersected the differentially expressed genes with our high‐throughput human CRISPR‐based Brunello knockout (KO) screen (Figure 1C and Table S3) [31]. The transcription factor MEF2B stood out with a fold change of −3.0 in the Brunello KO screen, indicating that knockdown of this gene has a strong negative effect on growth of ST486. Based on this, we selected MEF2B for further analysis. Downregulation of MEF2B upon ZDHHC11 knockdown in ST486 was confirmed at the protein level by Western blotting. A decrease of 60%–75% was observed for the main isoform of MEF2B (isoA) and of 75%–90% for the weaker, low molecular weight MEF2B isoform (isoB) (Figure 1D and S1 B). These effects were similar to those observed at the MEF2B RNA levels (Figure S1 A).

Knockdown of all *ZDHHC11* transcripts also showed a negative effect on growth of two other BL cell lines, BL‐41 and CA46 (Figure S1 C). Consistent with the lower knockdown efficiency, the effect on growth was weaker as compared to ST486 (Figure S1 D). Although mild effects were observed for ZDHHC11‐sh3 in BL‐41 and for ZDHHC11‐sh2 in CA46 on MEF2B RNA and protein levels, we were unable to show a clear downregulation (Figure S1 B,E). This is probably due to the suboptimal ZDHHC11 knockdown efficiency.

### 3.2 MEF2B Is Important for BL Cell Growth and Regulates BCL6 Expression in BL

**FIGURE 2:**
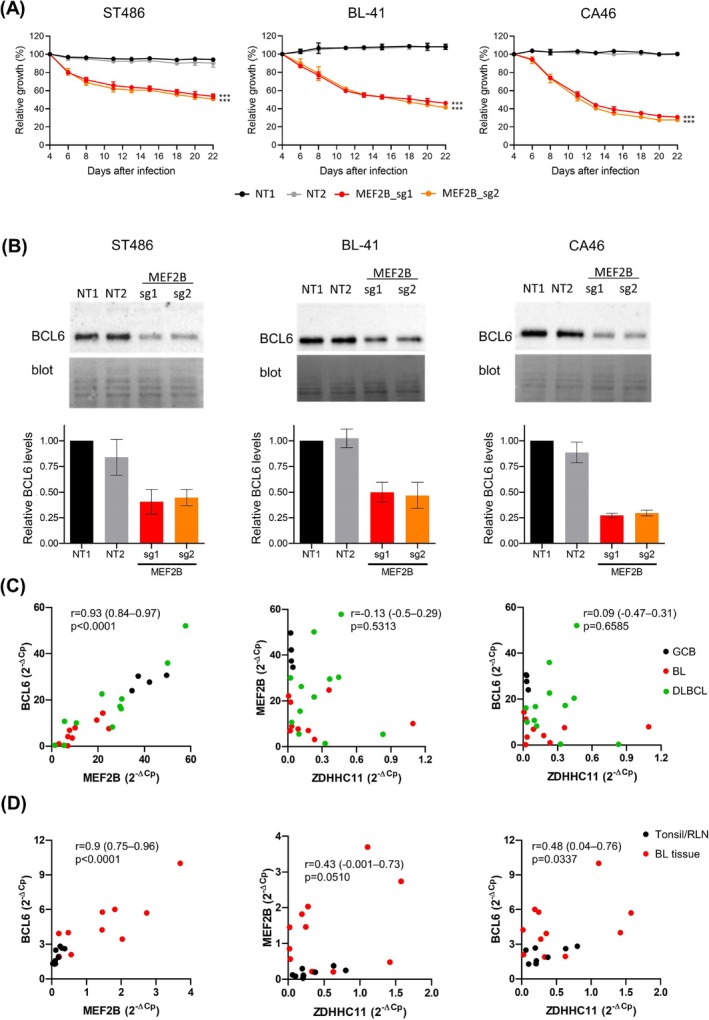
MEF2B is important for BL cell growth and regulates BCL6 expression in BL. ST486, BL‐41 and CA46 cells were transduced with lentiviral vectors carrying control (NT1: Black and NT2: Grey) or sgRNA sequences targeting MEF2B (sg1: Red and sg2: Orange). (A) Relative growth of BL cells upon MEF2B knockdown was measured by following the percentage of GFP+ cells over 3 weeks post‐transduction, with the percentages normalized to Day 4 after transduction. Mean ± SD of three independent experiments is shown. Significance was determined by mixed model analysis; ***p < 0.001. (B) Western blots show BCL6 protein levels upon MEF2B knockdown. Quantification was normalized to the total amount of protein loaded per lane, and the data were plotted relative to the NT1 control sample. A representative blot along with the mean ± SD of two independent biological experiments is shown, with each sample loaded twice on gel. (C) Correlation analysis between relative MEF2B, BCL6 and ZDHHC11 transcript levels in germinal center B‐cells sorted from tonsils (GCB, *n* = 4), BL (*n* = 9) and DLBCL (*n* = 12) cell lines as measured by RT‐qPCR. Pearson's r values (95% confidence interval) are given. (D) Same as (C) but in primary BL (*n* = 11) and control tissues (tonsils, *n* = 6 and reactive lymph nodes (RLN), *n* = 3).

To confirm the essentiality of MEF2B based on the Brunello KO screen, we determined the effect of MEF2B knockdown on growth of three BL cell lines. A clear decrease in MEF2B protein levels by 60%–85% was achieved using two independent single guide RNAs (sgRNAs) (Figure 2A). Knockdown of MEF2B resulted in strong inhibition of cell growth in all three BL cell lines (Figure 2A), with a decrease in GFP^+^ cells reaching 45%–50% at day 22 after transduction in ST486, 55%–60% for BL‐41, and 70% for CA46. The strength of the phenotype positively correlated with the endogenous MEF2B levels in the cell lines (Figure S2 B). Overall, these results show that MEF2B is important for growth of BL cells.

As MEF2B is a known regulator of BCL6 in normal GC B‐cells, DLBCL and FL, we next studied its effect on BCL6 in BL [22]. Knockdown of MEF2B repressed BCL6 expression at the RNA and protein level in three BL cell lines. RNA levels were repressed by 65%–70% in ST486 and CA46, and 40%–50% in BL‐41 (Figure S3 A). Protein levels were repressed by 50%–70% in the three cell lines (Figure 2B). Analysis of MEF2B and BCL6 mRNA levels in GC B‐cells, BL and DLBCL cell lines revealed a strong correlation between MEF2B and BCL6 mRNA levels (*r* = 0.93 [95% CI 0.84–0.97], *p* < 0.0001), consistent with the regulatory role of MEF2B for BCL6 (Figure 2C). MEF2B and BCL6 protein levels also correlated in BL cell lines (Figure S2 B,C). However, ZDHHC11 mRNA levels did not correlate with those of MEF2B nor BCL6 (Figure 2C).

Similar to the results observed in cell lines, MEF2B and BCL6 mRNA levels were also strongly correlated in primary BL and control tissues (*r* = 0.9 [95% CI 0.75–0.96], *p* < 0.0001). Moreover, ZDHHC11 levels showed a weak correlation with MEF2B (*r* = 0.43 [95% CI −0.01–0.73], *p* = 0.0510) and BCL6 (*r* = 0.48 [95% CI 0.04–0.76], *p* = 0.0337) (Figure 2D). Together, these results highlight a clear dependency between MEF2B and BCL6 in BL and suggest ZDHHC11 as an upstream factor involved in the regulation of the MEF2B‐BCL6 axis.

### 3.3 BCL6 Promotes BL Cell Growth

**FIGURE 3:**
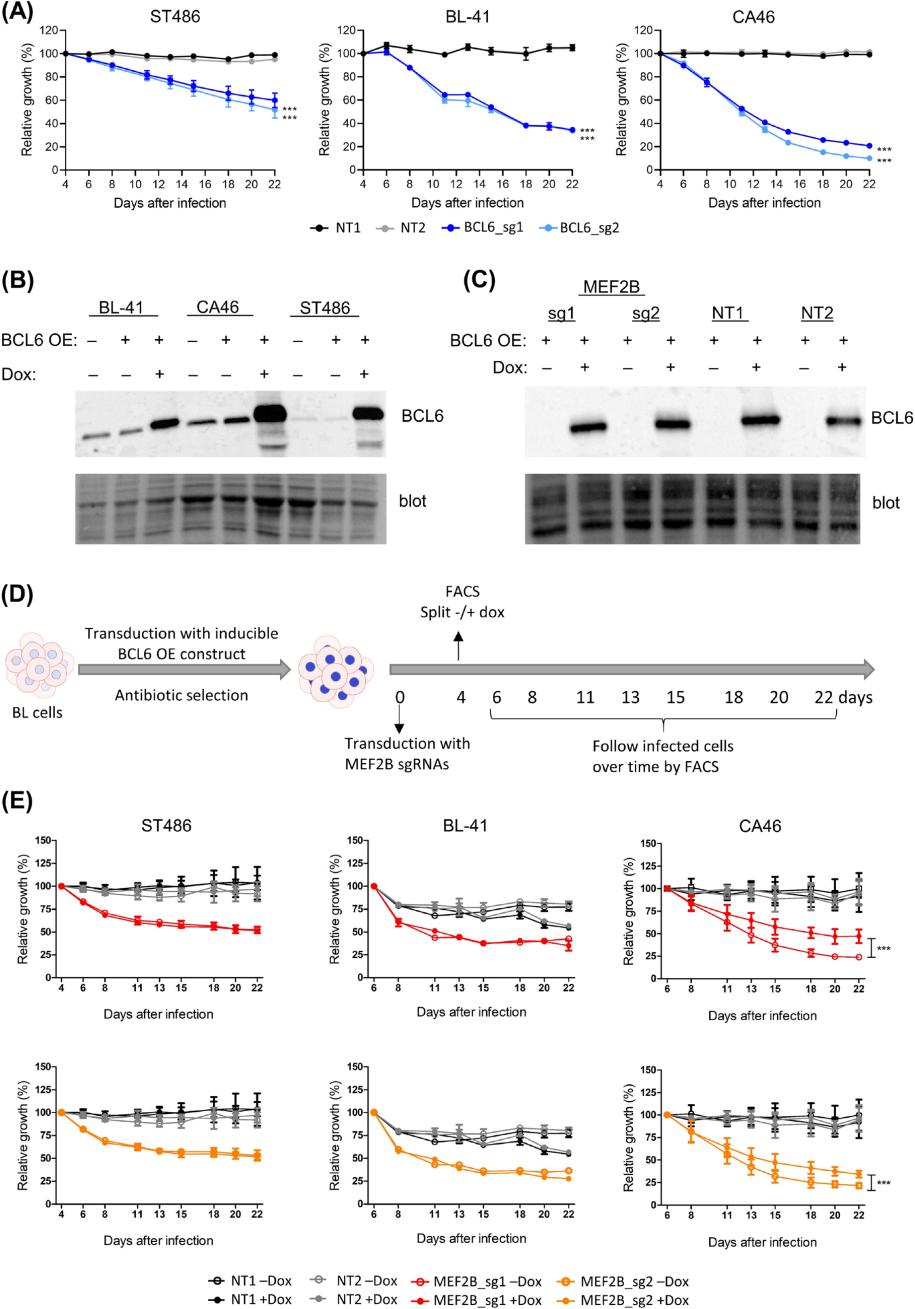
The growth‐supportive role of MEF2B in BL partly depends on BCL6. (A) ST486, BL‐41 and CA46 cells were transduced with lentiviral vectors carrying control (NT1: Black and NT2: Grey) or sgRNA sequences targeting BCL6 (sg1: Dark blue and sg2: Light blue). Relative growth of BL cells upon BCL6 knockdown was measured by following the percentage of GFP+ cells over 3 weeks post‐transduction, with the percentages normalized to Day 4 after transduction. Mean ± SD of three independent experiments is shown. Significance was determined by mixed model analysis; ***p < 0.001. (B‐C) ST486, BL‐41 and CA46 cells were transduced with a vector for a doxycycline‐inducible BCL6 overexpression (BCL6 OE). (B) Protein levels of BCL6 in WT cells, transduced cells without doxycycline and treated with 1 μg/mL doxycycline for 48 h (*N* = 1). (C) ST486 cells were transduced with sgRNAs targeting MEF2B or control NT vectors and cultured with 1 μg/mL or without doxycycline for 48 h (*N* = 1). (D) Timeline of the rescue experiment. (E) ST486, BL‐41 and CA46 cells were transduced with lentiviral vectors carrying control (NT1: Black and NT2: Grey) or sgRNA sequences targeting MEF2B (sg1: Red and sg2: Orange) and cultured with 1 μg/mL or without doxycycline. Relative growth of BL cells was measured by following the percentage of GFP+ cells over 3 weeks post‐transduction, with the percentages normalized to Day 4 or 6 after transduction. Mean ± SD of two independent experiments is shown. Significance was determined by mixed model analysis; ***p < 0.001.

Next, we tested the effect of BCL6 knockdown on BL cell growth. A clear knockdown of BCL6 was observed using two sgRNAs with a decrease in BCL6 protein levels of more than 90% in ST486 and CA46, and 80%–90% in BL‐41 (Figure S3 B). Downregulation of BCL6 resulted in strong inhibition of growth in three BL cell lines (Figure 3A). The reduction in GFP^+^ cells on day 22 post‐transduction was 40%–50% in ST486, 65% in BL‐41, and 80%–90% in CA46. Similar to MEF2B, the strength of the phenotype correlated with the endogenous BCL6 levels (Figure S2 C). These results show that BCL6 is also essential for BL cell growth.

### 3.4 The Growth‐Supportive Role of MEF2B in BL Partly Depends on BCL6

To determine how essential the regulation of BCL6 by MEF2B is for BL growth we performed a rescue experiment. Using a tetracycline‐inducible system we achieved a strong overexpression of BCL6 in all cell lines (Figure 3B). Similar BCL6 overexpression levels were achieved in MEF2B KD and control sgRNA treated cells (Figure 3C). Combination of BCL6 overexpression with knockdown of the endogenous BCL6 mitigated the growth suppressive effect of BCL6 knockdown, confirming functional overexpression of BCL6 (Figure S4). GFP competition assays with MEF2B sgRNAs in parallel with and without BCL6 induction revealed a consistent significant partial rescue of the phenotype in CA46, i.e., 30% rescue for MEF2B sgRNA1 and 15% for sgRNA2 (Figure 3D,E). However, BCL6 overexpression did not rescue the effect of MEF2B knockdown in ST486 and BL‐41 cells (Figure 3E). These results suggest that MEF2B regulates multiple crucial growth‐supportive genes including BCL6 in BL.

## 4 Discussion

In the present study we investigated the role of the MEF2B‐BCL6 axis in Burkitt lymphoma. MEF2B and BCL6 are critical factors in GC B cell function and lymphomagenesis [12, 21, 23]. High expression of MEF2B as well as frequent mutations in the gene have been shown in DLBCL and FL but no mutations have been reported in BL [12, 22, 32, 33]. While the MEF2B‐BCL6 relationship has been clearly defined in other B‐cell lymphomas, the mechanism of MEF2B deregulation and its significance for BL cell survival has not been investigated before. Here we show a potential link between MEF2B and ZDHHC11 and demonstrate the importance of ZDHHC11, MEF2B and BCL6 for BL cell growth. Despite showing a clear regulatory role of MEF2B on BCL6 expression in BL, growth inhibition induced by MEF2B knockdown could only partly be rescued by restoration of BCL6 expression.

Based on our previously published study, we proposed that ZDHHC11 transcripts support BL proliferation by sequestering miR‐150, thereby relieving its inhibitory effect on MYB, a transcription factor essential for the high proliferation rate of BL cells [24]. However, our recent work revealed that the growth‐supporting function of ZDHHC11 in BL is not limited to miR‐150 sequestration. Monoclonal cell lines with targeted deletion of the ZDHHC11 miR‐150 binding site region were viable and showed normal population doubling times. Moreover, knockdown of circZDHHC11 in BL cells lacking the miR‐150 binding region still led to impaired cell proliferation [26]. These results indicate a miR‐150‐independent role for ZDHHC11 and suggest that this region is not essential for the growth‐supporting role of ZDHHC11. We previously showed that knockdown of circZDHHC11 did not affect global gene expression [26]. Together, this suggests that the linear transcripts (protein coding and/or long noncoding) are more likely to be involved in the here observed effects. The effect of ZDHHC11 KD was most prominent in ST486 and less prominent in two other BL cell lines, probably due to a much lower KD efficiency. MEF2B showed a borderline significant correlation with ZDHHC11 levels in a cohort of BL patients, which suggests a potential regulatory link. Thus, our findings point to an alternative growth‐supportive mechanism of ZDHHC11 via downstream effectors, including MEF2B. To further support our findings, in vivo experiments are needed. Whether the mechanism by which ZDHHC11 regulates MEF2B is direct or indirect remains to be determined. A potential mechanism might be that stability and localization of MEF2B are modulated by ZDHHC11 However, there is currently no evidence that MEF2B can be modified by S‐palmitoylation. Alternatively, the ZDHHC11 long noncoding RNA could affect MEF2B expression via transcriptional regulation.

We showed that KD of MEF2B and BCL6 both negatively affected survival of BL cells, albeit with some phenotypic differences between cell lines. These might be related to differences in endogenous levels of MEF2B and BCL6 as well as differences in their genomic landscapes, chromatin state, co‐factor interactions and survival pathways. Decrease in cell growth upon MEF2B KD was the strongest in CA46 and weakest in ST486, and this correlated with MEF2B protein levels. The same pattern was observed for BCL6 KD, which supports the link between MEF2B and BCL6 in BL. BCL6 overexpression partly rescued growth inhibition caused by MEF2B KD only in CA46 which showed the strongest phenotype upon BCL6 depletion. We hypothesize that BCL6 is a critical target of MEF2B in CA46, while next to BCL6, several other essential targets are regulated by MEF2B in the other two BL cell lines. This can explain why BCL6 overexpression was not sufficient to recover the transcriptional pathways required for optimal BL proliferation.

The association between MEF2B and BCL6 expression was first observed in DLBCL using immunohistochemistry by Krenács et al. [22]. Further studies indeed showed that MEF2B is a transcriptional regulator of BCL6 in DLBCL. However, the details of the interaction between MEF2B and BCL6, the relationship between MEF2B and other GC B‐cell markers, as well as its role in other B‐cell lymphomas such as BL have not yet been fully elucidated [12, 34]. In DLBCL cell lines, MEF2B was demonstrated to function as a transcriptional co‐activator of BCL6, acting in cooperation with AP‐2α. MEF2B knockdown reduced BCL6 and CD10 levels, decreased ERK1/2 phosphorylation, and impaired lymphoma cell growth, especially in GC‐DLBCL lines where MEF2B is preferentially expressed [12]. Our findings in BL cell lines and patient samples also indicate a correlation between MEF2B and BCL6 levels. In addition, MEF2B knockdown significantly reduced BCL6 levels in all BL cell lines and downregulation of either MEF2B or BCL6 led to a similar strong growth impairment. This suggested that MEF2B may exert its growth supporting effects in BL via BCL6. BCL6 overexpression partly restored proliferation in MEF2B‐depleted CA46 cells, but not in two other BL cell lines. Similar observations were made in DLBCL, where BCL6 knockdown resulted in decreased proliferation but enforced expression of BCL6 alone did not rescue the impaired proliferation upon MEF2B knockdown [12]. Thus, MEF2B activation likely induces multiple factors, including BCL6, that are essential for lymphoma growth. The results of BCL6 KD in BL are in line with previous work that showed that BCL6 is a critical factor in lymphomagenesis, as supported by its recurrent translocations in DLBCL and FL and functional validation in transgenic mice models [35]. Interestingly, mutations in MEF2B were reported as an alternative way of BCL6 activation, complementing previously reported mechanisms such as promoter substitution caused by chromosomal translocations, mutations in the BCL6 promoter region, or prevention of BCL6 degradation due to inactivating mutations in the ubiquitin ligase FBXO1 [12, 36, 37, 38, 39]. Moreover, BCL6 rearrangements seem to be mutually exclusive with activating mutations of MEF2B in DLBCL, implying that both type of mutations function in the same oncogenic axis [33]. Overall, our results in BL, as well as previously published data in DLBCL, underscore that the role of MEF2B extends beyond BCL6, supporting the hypothesis that MEF2B coordinates a broader transcriptional program that supports lymphomagenesis.

So far, no direct target genes of MEF2B other than BCL6 have been confirmed in B‐cell lymphoma. The most relevant study in GC B cells revealed 250 genes bound by MEF2B and differentially expressed in human and mouse upon MEFB modulation. Identified MEF2B target genes were involved in processes such as cell cycle, apoptosis, DNA replication and repair, or chromatin remodeling [21]. This included genes identified as essential for ST486 cell survival in a previously conducted sgRNA dropout screen, such as CCND3, MCM3, RFC3, and BCL2L1 [31]. Insights into MEF2B function revealed its interaction with the SWI/SNF chromatin‐remodeling complex, which is modulated by MEF2B S324 phosphorylation. 50% of MEF2B‐bound regions in normal human GC B cells, corresponding to 5661 genes, were co‐occupied by SMARCA4, a subunit of the SWI/SNF complex. Pathway enrichment analyses revealed processes such as cell cycle, DNA repair, apoptosis, and GC confinement [34]. Another study in HEK293 cells identified 1141 candidate direct target genes of MEF2B, which were implicated in cell movement and survival [40].

In summary, our findings expand the growing knowledge of transcriptional regulation in BL and highlight the previously unrecognized ZDHHC11–MEF2B–BCL6 regulatory axis. Although MEF2B and BCL6 proteins have established functions in GC B‐cell biology and lymphomagenesis, the role of MEF2B in BL has not been investigated before. Our work highlights MEF2B as a crucial transcription factor in BL and suggests ZDHHC11 as a potential upstream regulator of MEF2B. We confirmed BCL6 as an important downstream target of MEF2B with a growth supporting role in BL, but our data indicate that there are other critical MEF2B target genes. In order to enhance the biological relevance and translational impact of this study, future research should focus on unraveling the full transcriptional network of MEF2B and identifying the complete spectrum of crucial target genes, as well as on in vivo validation of the findings.

## Author Contributions

**Lotteke J. Y. M. Ziel‐Swier:** conceptualization, methodology, investigation, writing – original draft, visualization; **Karolina Rassek:** methodology, investigation, writing – original draft, visualization; **Yichen Liu:** investigation, visualization, funding acquisition; **Annika Seitz, Jasper Koerts, Debora de Jong, Bea Rutgers and Julia Przybył:** investigation; **Martine E. D. Chamuleau:** resources; **Anke van den Berg:** conceptualization, supervision, funding acquisition, writing – review and editing; **Agnieszka Dzikiewicz‐Krawczyk:** conceptualization, supervision, formal analysis, visualization, witing – review and editing; **Joost Kluiver:** conceptualization, supervision, formal analysis, funding acquisition, writing – review and editing.

## Funding

This work was supported by the grants from Lymph&Co (AvdB and JK), Zeldzame Ziekten Fonds (ZZF) (JK), the China Scholarship Council (CSC) (YL) and H2020 Spreading Excellence and Widening Participation Grant Number: 952304 (AD‐K and AvdB). KR was supported by the Foundation for Polish Science (FNP).

## Ethics Statement

Written permission for the use of tonsil tissues to isolate GC B‐cells was obtained from the parents of the children. The protocol was consistent with the international ethical guidelines (Declaration of Helsinki and the International Conference on Harmonization Guidelines for Good Clinical Practice).

The study utilized residual material from patients (BL tissues), the use of which is regulated under the code for good clinical practice in the Netherlands and does not require informed consent in accordance with Dutch regulations and is in compliance with national ethical guidelines (“Code for Proper Secondary Use of Human Tissue,” Dutch Federation of Medical Scientific Societies) and the declaration of Helsinki.

## Conflicts of Interest

The authors declare no conflicts of interest.

## Supporting information


**Figure S1:** Efficiency and effect of ZDHHC11 knockdown. ST486, BL‐41 and CA46 cells were transfected with lentiviral vectors carrying control (NT1: black and NT2: grey) or shRNA sequences targeting all three ZDHHC11 transcripts (sh1: dark green and sh2: light green). (A) Validation of 10 putative ZDHHC11 targets by qRT PCR in ST486 cells transfected with control or ZDHHC11_all shRNAs. Mean with error bars of one experiment performed in triplo is shown, and the data were normalized to the NT1 control. (B) Western blots show MEF2B levels upon ZDHHC11 knockdown. Quantification of both the upper, most prominent MEF2B bands and the lower, very weak MEF2B bands was normalized to the total amount of protein loaded per lane, and the data were plotted relative to the NT1 control sample. A representative blot and mean ± SD of two independent experiments is shown, with each sample loaded twice on gel. (C) Relative growth of BL‐41 and CA46 cells upon ZDHHC11 knockdown was measured by following the percentage of GFP+ cells over 3 weeks post‐transduction, with the percentages normalized to day 4 after transduction. Mean ± SD of three independent experiments is shown. Significance was determined by mixed model analysis; ***p < 0.001 (D–E). Relative expression of all ZDHHC11 transcripts (D) and MEF2B (e) upon knockdown of the ZDHHC11 transcripts was measured by qRT‐PCR. Mean ± SD of two independent experiments is shown and the data were normalized to the NT1 control.
**Figure S2:** Efficiency of MEF2B knockdown and endogenous MEF2B and BCL6 levels. (A) ST486, BL‐41 and CA46 cells were transduced with lentiviral vectors carrying control (NT1: black and NT2: grey) or sgRNA sequences targeting MEF2B (sg1: red and sg2: orange). Western blots show downregulation of MEF2B levels upon MEF2B knockdown in the four BL cell lines. Quantification of the upper, stronger, and lower, weaker MEF2B bands was normalized to the total amount of protein loaded per lane, and the data were plotted relative to the NT1 control sample. A representative blot along with the mean ± SD of two independent experiments is shown, with each sample loaded twice on gel. Western blots show endogenous MEF2B (B) and BCL6 (C) levels in BL cell lines. Quantification was normalized to the total amount of protein loaded per lane, and the data were plotted relative to the levels in CA46. A representative blot along with the mean ± SD of two independent experiments is shown, with each sample loaded twice on gel. X—positive control.
**Figure S3:** BCL6 expression in BL cell lines upon MEF2B or BCL6 knockdown. (A) ST486, BL‐41 and CA46 cells were transduced with lentiviral vectors carrying control (NT1: black and NT2: grey) or sgRNA sequences targeting MEF2B (sg1: red and sg2: orange). Relative BCL6 RNA levels upon knockdown of MEF2B were measured by qRT‐PCR. Mean ± SD of two independent experiments is shown, and the data were normalized to the NT1 control. (B) ST486, BL‐41 and CA46 cells were transduced with lentiviral vectors carrying control (NT1: black and NT2: grey) or sgRNA sequences targeting BCL6 (sg1: dark blue and sg2: light blue). Western blots show BCL6 protein levels upon BCL6 knockdown. Quantification was normalized to the total amount of protein loaded per lane, and the data were plotted relative to the NT1 control sample. A representative blot along with the mean ± SD of two independent experiments is shown, with each sample loaded twice on gel.
**Table S1:** Sequences of shRNAs, sgRNAs and qRT‐PCR primers.
**Table S2:** Antibodies used in Western blotting.
**Table S3:** Probes up‐ or downregulated upon knockdown of all ZDHHC11 transcripts. Probes were found to be differentially expressed upon knockdown of all ZDHHC11 transcripts with FC ≥ 2.0. The order is the same as the hierarchical clustering shows in Figure 1. FC and *p* value of the microarray and a previously performed high‐throughput screen with the human CRISPR Brunello knockout library performed in ST486 are presented.

## Data Availability

The raw Agilent SurePrint G3 Human GE 8 × 60 microarray data generated in this study are available in GEO under accession number GSE218286. Other data that support the findings of this study are available from the corresponding author upon request.

## References

[1] J. Shingleton , J. Wang , C. Baloh , et al., “Non‐Hodgkin Lymphomas: Malignancies Arising From Mature B Cells,” Cold Spring Harbor Perspectives in Medicine 11 (2021): a034843. 32152246 10.1101/cshperspect.a034843 PMC7919396

[2] E. M. Molyneux , R. Rochford , B. Griffin , et al., “Burkitt's Lymphoma,” Lancet 379 (2012): 1234–1244. 22333947 10.1016/S0140-6736(11)61177-X

[3] C. Lopez , K. Kleinheinz , S. M. Aukema , et al., “Genomic and Transcriptomic Changes Complement Each Other in the Pathogenesis of Sporadic Burkitt Lymphoma,” Nature Communications 10 (2019): 1459. 10.1038/s41467-019-08578-3 PMC6440956 30926794

[4] Z. Y. Xu‐Monette , Q. Deng , G. C. Manyam , et al., “Clinical and Biologic Significance of MYC Genetic Mutations in De Novo Diffuse Large B‐Cell Lymphoma,” Clinical Cancer Research 22 (2016): 3593–3605. 26927665 10.1158/1078-0432.CCR-15-2296 PMC4947447

[5] E. Kumar , L. Pickard , and J. Okosun , “Pathogenesis of Follicular Lymphoma: Genetics to the Microenvironment to Clinical Translation,” British Journal of Haematology 194 (2021): 810–821. 33694181 10.1111/bjh.17383

[6] J. M. Schuetz , N. A. Johnson , R. D. Morin , et al., “BCL2 Mutations in Diffuse Large B‐Cell Lymphoma,” Leukemia 26 (2012): 1383–1390. 22189900 10.1038/leu.2011.378

[7] J. Zhang , D. Dominguez‐Sola , S. Hussein , et al., “Disruption of KMT2D Perturbs Germinal Center B Cell Development and Promotes Lymphomagenesis,” Nature Medicine 21 (2015): 1190–1198. 10.1038/nm.3940 PMC5145002 26366712

[8] J. Zhang , S. Vlasevska , V. A. Wells , et al., “The CREBBP Acetyltransferase Is a Haploinsufficient Tumor Suppressor in B‐Cell Lymphoma,” Cancer Discovery 7 (2017): 322–337. 28069569 10.1158/2159-8290.CD-16-1417 PMC5386396

[9] R. D. Morin , N. A. Johnson , T. M. Severson , et al., “Somatic Mutations Altering EZH2 (Tyr641) in Follicular and Diffuse Large B‐Cell Lymphomas of Germinal‐Center Origin,” Nature Genetics 42 (2010): 181–185. 20081860 10.1038/ng.518 PMC2850970

[10] L. K. Hilton , B. Collinge , S. Ben‐Neriah , et al., “Motive and Opportunity: MYC Rearrangements in High‐Grade B‐Cell Lymphoma With MYC and BCL2 Rearrangements (An LLMPP Study),” Blood 144 (2024): 525–540. 38701426 10.1182/blood.2024024251 PMC11307266

[11] R. Alsharif and K. Dunleavy , “Burkitt Lymphoma and Other High‐Grade B‐Cell Lymphomas With or Without MYC, BCL2, and/or BCL6 Rearrangements,” Hematology/Oncology Clinics of North America 33 (2019): 587–596. 31229156 10.1016/j.hoc.2019.04.001

[12] C. Y. Ying , D. Dominguez‐Sola , M. Fabi , et al., “MEF2B Mutations Lead to Deregulated Expression of the Oncogene BCL6 in Diffuse Large B Cell Lymphoma,” Nature Immunology 14 (2013): 1084–1092. 23974956 10.1038/ni.2688 PMC3954820

[13] A. Aziz , Q. C. Liu , and F. J. Dilworth , “Regulating a Master Regulator: Establishing Tissue‐Specific Gene Expression in Skeletal Muscle,” Epigenetics 5 (2010): 691–695. 20716948 10.4161/epi.5.8.13045 PMC3052885

[14] K. Cante‐Barrett , R. Pieters , and J. P. Meijerink , “Myocyte Enhancer Factor 2C in Hematopoiesis and Leukemia,” Oncogene 33 (2014): 403–410. 23435431 10.1038/onc.2013.56

[15] C. A. Desjardins and F. J. Naya , “The Function of the MEF2 Family of Transcription Factors in Cardiac Development, Cardiogenomics, and Direct Reprogramming,” Journal of Cardiovascular Development and Disease 3 (2016): 26. 27630998 10.3390/jcdd3030026 PMC5019174

[16] R. I. Clark , S. W. S. Tan , C. B. Péan , et al., “MEF2 Is an In Vivo Immune‐Metabolic Switch,” Cell 155 (2013): 435–447. 24075010 10.1016/j.cell.2013.09.007 PMC3807682

[17] V. Prima and S. P. Hunger , “Cooperative Transformation by MEF2D/DAZAP1 and DAZAP1/MEF2D Fusion Proteins Generated by the Variant t(1;19) in Acute Lymphoblastic Leukemia,” Leukemia 21 (2007): 2470–2475. 17898785 10.1038/sj.leu.2404962

[18] M. Schwieger , A. Schüler , M. Forster , et al., “Homing and Invasiveness of MLL/ENL Leukemic Cells Is Regulated by MEF2C,” Blood 114 (2009): 2476–2488. 19584403 10.1182/blood-2008-05-158196

[19] E. Di Giorgio , A. Clocchiatti , S. Piccinin , et al., “MEF2 Is a Converging Hub for Histone Deacetylase 4 and Phosphatidylinositol 3‐Kinase/Akt‐Induced Transformation,” Molecular and Cellular Biology 33 (2013): 4473–4491. 24043307 10.1128/MCB.01050-13 PMC3838174

[20] M. Saito , J. Gao , K. Basso , et al., “A Signaling Pathway Mediating Downregulation of in Germinal Center B Cells Is Blocked by Gene Alterations in B Cell Lymphoma (Vol 12, Pg 280, 2007),” Cancer Cell 12 (2007): 280–292. 17785208 10.1016/j.ccr.2007.08.011

[21] P. Brescia , C. Schneider , A. B. Holmes , et al., “MEF2B Instructs Germinal Center Development and Acts as an Oncogene in B Cell Lymphomagenesis,” Cancer Cell 34 (2018): 453–465. 30205047 10.1016/j.ccell.2018.08.006 PMC6223119

[22] D. Krenacs , Z. Borbenyi , J. Bedekovics , G. Mehes , E. Bagdi , and L. Krenacs , “Pattern of MEF2B Expression in Lymphoid Tissues and in Malignant Lymphomas,” Virchows Archiv 467 (2015): 345–355. 26089142 10.1007/s00428-015-1796-6

[23] S. M. El Jamal , Z. Grada , M. H. El Dinali , et al., “MEF2B Is a Member of the Gene Transcriptional Complex and Induces Its Expression in Diffuse Large B‐Cell Lymphoma of the Germinal Center B‐Cell‐Like Type,” Laboratory Investigation 99 (2019): 539–550. 30446717 10.1038/s41374-018-0152-2

[24] A. Dzikiewicz‐Krawczyk , K. Kok , I. Slezak‐Prochazka , et al., “ZDHHC11 and ZDHHC11B Are Critical Novel Components of the Oncogenic MYC‐miR‐150‐MYB Network in Burkitt Lymphoma,” Leukemia 31 (2017): 1470–1473. 28331227 10.1038/leu.2017.94

[25] K. Lemonidis , M. W. Werno , J. Greaves , et al., “The zDHHC Family of S‐Acyltransferases,” Biochemical Society Transactions 43 (2015): 217–221. 25849920 10.1042/BST20140270

[26] Y. Liu , X. Zhao , A. Seitz , et al., “Circular ZDHHC11 Supports Burkitt Lymphoma Growth Independent of Its miR‐150 Binding Capacity,” Scientific Reports 14 (2024): 8730. 38627588 10.1038/s41598-024-59443-3 PMC11021472

[27] L. Ziel‐Swier , Y. Liu , A. Seitz , et al., “The Role of the MYC/miR‐150/MYB/ZDHHC11 Network in Hodgkin Lymphoma and Diffuse Large B‐Cell Lymphoma,” Genes (Basel) 13 (2022): 227. 35205272 10.3390/genes13020227 PMC8871936

[28] Y. Yuan , J. Kluiver , J. Koerts , et al., “miR‐24‐3p Is Overexpressed in Hodgkin Lymphoma and Protects Hodgkin and Reed‐Sternberg Cells From Apoptosis,” American Journal of Pathology 187 (2017): 1343–1355. 28432871 10.1016/j.ajpath.2017.02.016

[29] C. MED , F. Stenner , D. A. Chitu , et al., “R‐CODOX‐M/R‐IVAC Versus DA‐EPOCH‐R in Patients With Newly Diagnosed Burkitt Lymphoma (HOVON/SAKK): Final Results of a Multicentre, Phase 3, Open‐Label, Randomised Trial,” Lancet Haematology 10 (2023): e966–e975. 37922925 10.1016/S2352-3026(23)00279-X

[30] D. M. Walter , O. S. Venancio , E. L. Buza , et al., “Systematic In Vivo Inactivation of Chromatin‐Regulating Enzymes Identifies Setd2 as a Potent Tumor Suppressor in Lung Adenocarcinoma,” Cancer Research 77 (2017): 1719–1729. 28202515 10.1158/0008-5472.CAN-16-2159 PMC5380596

[31] M. Kazimierska , M. Podralska , M. Zurawek , et al., “CRISPR/Cas9 Screen for Genome‐Wide Interrogation of Essential MYC‐Bound E‐Boxes in Cancer Cells,” Molecular Oncology 17 (2023): 2295–2313. 37519063 10.1002/1878-0261.13493 PMC10620128

[32] E. M. Moore , S. H. Swerdlow , and S. E. Gibson , “Comparison of Myocyte Enhancer Factor 2B Versus Other Germinal Center‐Associated Antigens in the Differential Diagnosis of B‐Cell Non‐Hodgkin Lymphomas,” American Journal of Surgical Pathology 42 (2018): 342–350. 29309299 10.1097/PAS.0000000000001015

[33] R. D. Morin , M. Mendez‐Lago , A. J. Mungall , et al., “Frequent Mutation of Histone‐Modifying Genes in Non‐Hodgkin Lymphoma,” Nature 476 (2011): 298–303. 21796119 10.1038/nature10351 PMC3210554

[34] C. Yu , Q. Shen , A. B. Holmes , et al., “MEF2B C‐Terminal Mutations Enhance Transcriptional Activity and Stability to Drive B Cell Lymphomagenesis,” Nature Communications 15 (2024): 7195. 10.1038/s41467-024-51644-8 PMC11343756 39179580

[35] G. Cattoretti , L. Pasqualucci , G. Ballon , et al., “Deregulated BCL6 Expression Recapitulates the Pathogenesis of Human Diffuse Large B Cell Lymphomas in Mice,” Cancer Cell 7 (2005): 445–455. 15894265 10.1016/j.ccr.2005.03.037

[36] B. H. Ye , S. Chaganti , C. C. Chang , et al., “Chromosomal Translocations Cause Deregulated BCL6 Expression by Promoter Substitution in B Cell Lymphoma,” EMBO Journal 14 (1995): 6209–6217. 8557040 10.1002/j.1460-2075.1995.tb00311.x PMC394745

[37] L. Pasqualucci , A. Migliazza , K. Basso , J. Houldsworth , C. RSK , and R. Dalla‐Favera , “Mutations of the Proto‐Oncogene Disrupt Its Negative Autoregulation in Diffuse Large B‐Cell Lymphoma,” Blood 101 (2003): 2914–2923. 12515714 10.1182/blood-2002-11-3387

[38] S. Duan , L. Cermak , J. K. Pagan , et al., “FBXO11 Targets BCL6 for Degradation and Is Inactivated in Diffuse Large B‐Cell Lymphomas,” Nature 481 (2012): 90–93. 22113614 10.1038/nature10688 PMC3344385

[39] C. Pighi , T. C. Cheong , M. Compagno , et al., “Frequent Mutations of FBXO11 Highlight BCL6 as a Therapeutic Target in Burkitt Lymphoma,” Blood Advances 5 (2021): 5239–5257. 34625792 10.1182/bloodadvances.2021005682 PMC9153037

[40] J. R. Pon , J. Wong , S. Saberi , et al., “MEF2B Mutations in Non‐Hodgkin Lymphoma Dysregulate Cell Migration by Decreasing MEF2B Target Gene Activation,” Nature Communications 6 (2015): 7953. 10.1038/ncomms8953 PMC4918335 26245647

