# Supplementary material for: The Growth Supporting Role of ZDHHC11 Is Linked to the MEF2B–BCL6 Regulatory Circuit in Burkitt Lymphoma
Source: Int J Cancer. 2026 Apr 16;159(6):1535–45. doi: 10.1002/ijc.70506 (PMC13397227; doi:10.1002/ijc.70506)
Supplement: Supplementary file 1 — Figure S1: Efficiency and effect of ZDHHC11 knockdown. ST486, BL‐41 and CA46 cells were transfected with lentiviral vectors carrying control (NT1: black and NT2: grey) or shRNA sequences targeting all three ZDHHC11 transcripts (sh1: dark green and sh2: light green). (A) Validation of 10 putative ZDHHC11 targets by qRT PCR in ST486 cells transfected with control or ZDHHC11_all shRNAs. Mean with error bars of one experiment performed in triplo is shown, and the data were normalized to the NT1 control. (B) Western blots show MEF2B levels upon ZDHHC11 knockdown. Quantification of both the upper, most prominent MEF2B bands and the lower, very weak MEF2B bands was normalized to the total amount of protein loaded per lane, and the data were plotted relative to the NT1 control sample. A representative blot and mean ± SD of two independent experiments is shown, with each sample loaded twice on gel. (C) Relative growth of BL‐41 and CA46 cells upon ZDHHC11 knockdown was measured by following the percentage of GFP+ cells over 3 weeks post‐transduction, with the percentages normalized to day 4 after transduction. Mean ± SD of three independent experiments is shown. Significance was determined by mixed model analysis; ***p < 0.001 (D–E). Relative expression of all ZDHHC11 transcripts (D) and MEF2B (e) upon knockdown of the ZDHHC11 transcripts was measured by qRT‐PCR. Mean ± SD of two independent experiments is shown and the data were normalized to the NT1 control. Figure S2: Efficiency of MEF2B knockdown and endogenous MEF2B and BCL6 levels. (A) ST486, BL‐41 and CA46 cells were transduced with lentiviral vectors carrying control (NT1: black and NT2: grey) or sgRNA sequences targeting MEF2B (sg1: red and sg2: orange). Western blots show downregulation of MEF2B levels upon MEF2B knockdown in the four BL cell lines. Quantification of the upper, stronger, and lower, weaker MEF2B bands was normalized to the total amount of protein loaded per lane, and the data were plotted relative to [file IJC-159-1535-s001.pdf]

The growth supporting role of ZDHHC11 is linked to the MEF2B–BCL6 Regulatory Circuit in Burkitt Lymphoma

Lotteke J.Y.M Ziel-Swier, Karolina Rassek, Yichen Liu, Annika Seitz, Jasper Koerts, Debora de Jong, Bea Rutgers, Anastasiia Kompaniiets, Julia Przybył, Martine E.D. Chamuleau, Anke van den Berg, Agnieszka Dzikiewicz-Krawczyk, Joost Kluiver

Supplementary Materials

Supplementary Figures 1-4

Supplementary Tables 1-3

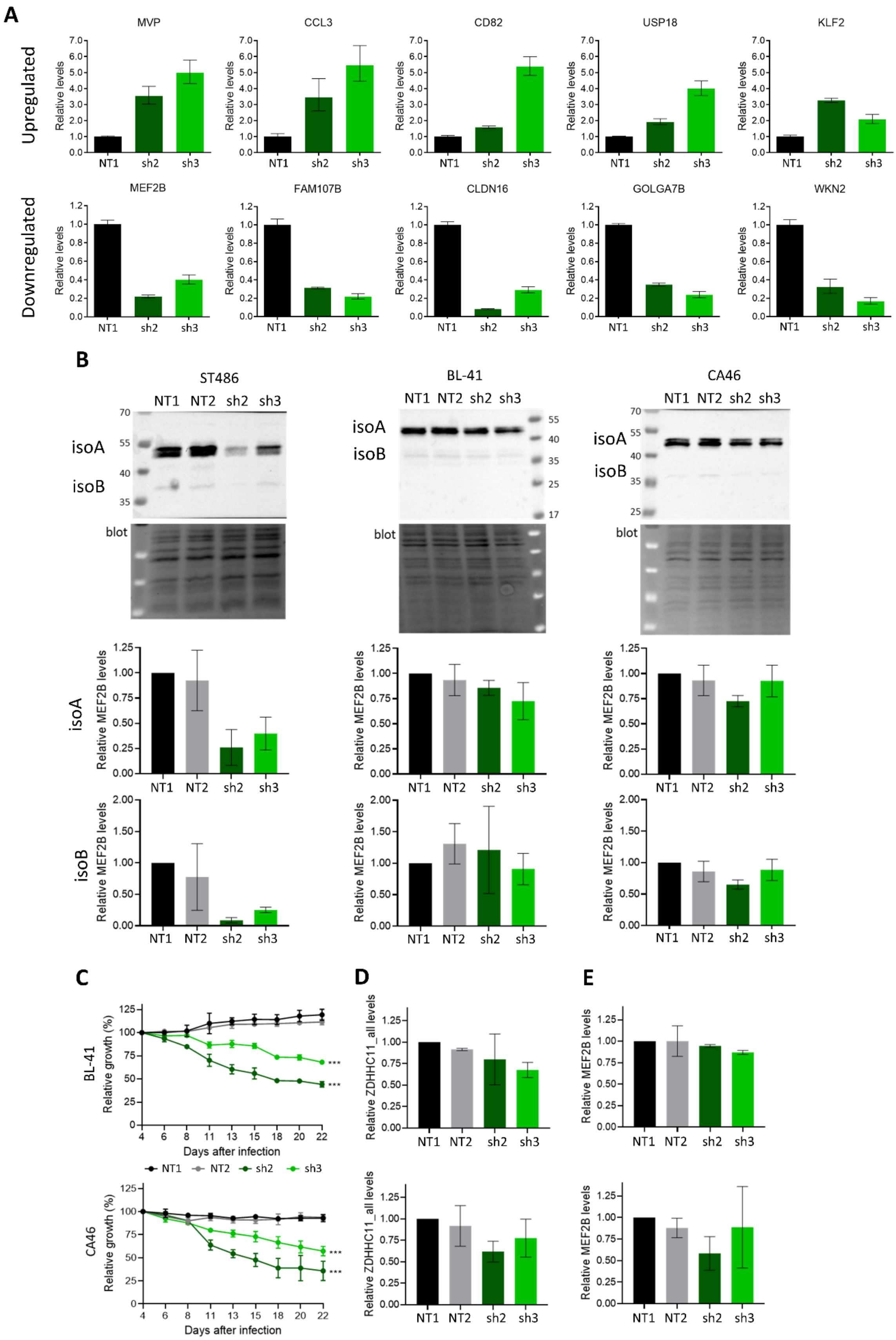
A
Upregulated
Downregulated
MVP
CCL3
CD82
USP18
KLF2
MEF2B
FAM107B
CLDN16
GOLGA7B
WKN2
Relative levels
NT1 sh2 sh3
B
ST486
BL-41
CA46
NT1 NT2 sh2 sh3
isoA
isoB
blot
isoA
isoB
Relative MEF2B levels
C
BL-41
CA46
Relative growth (%)
Days after infection
NT1 NT2 sh2 sh3
D
Relative ZDHHC11_all levels
E
Relative MEF2B levels

Supplementary Figure 1. Efficiency and effect of ZDHHC11 knockdown. ST486, BL-41 and CA46 cells were transfected with lentiviral vectors carrying control (NT1: black, NT2: grey) or shRNA sequences targeting all three ZDHHC11 transcripts (sh1: dark green, sh2: light green). (A) Validation of 10 putative ZDHHC11 targets by qRT-PCR in ST486 cells transfected with control or ZDHHC11_all shRNAs. Mean with error bars of one experiment performed in triplo is shown, and the data were normalized to the NT1 control. (B) Western blots show MEF2B levels upon ZDHHC11 knockdown. Quantification of both the upper, most prominent MEF2B bands and the lower, very weak MEF2B bands was normalized to the total amount of protein loaded per lane, and the data were plotted relative to the NT1 control sample. A representative blot and mean ± SD of two independent experiments is shown, with each sample loaded twice on gel. (C) Relative growth of BL-41 and CA46 cells upon ZDHHC11 knockdown was measured by following the percentage of GFP+ cells over three weeks post-transduction, with the percentages normalized to day 4 after transduction. Mean ± SD of three independent experiments is shown. Significance was determined by mixed model analysis; *** $p<0.001$. (D-E) Relative expression of all ZDHHC11 transcripts (D) and MEF2B (e) upon knockdown of the ZDHHC11 transcripts was measured by qRT-PCR. Mean ± SD of two independent experiments is shown and the data were normalized to the NT1 control.

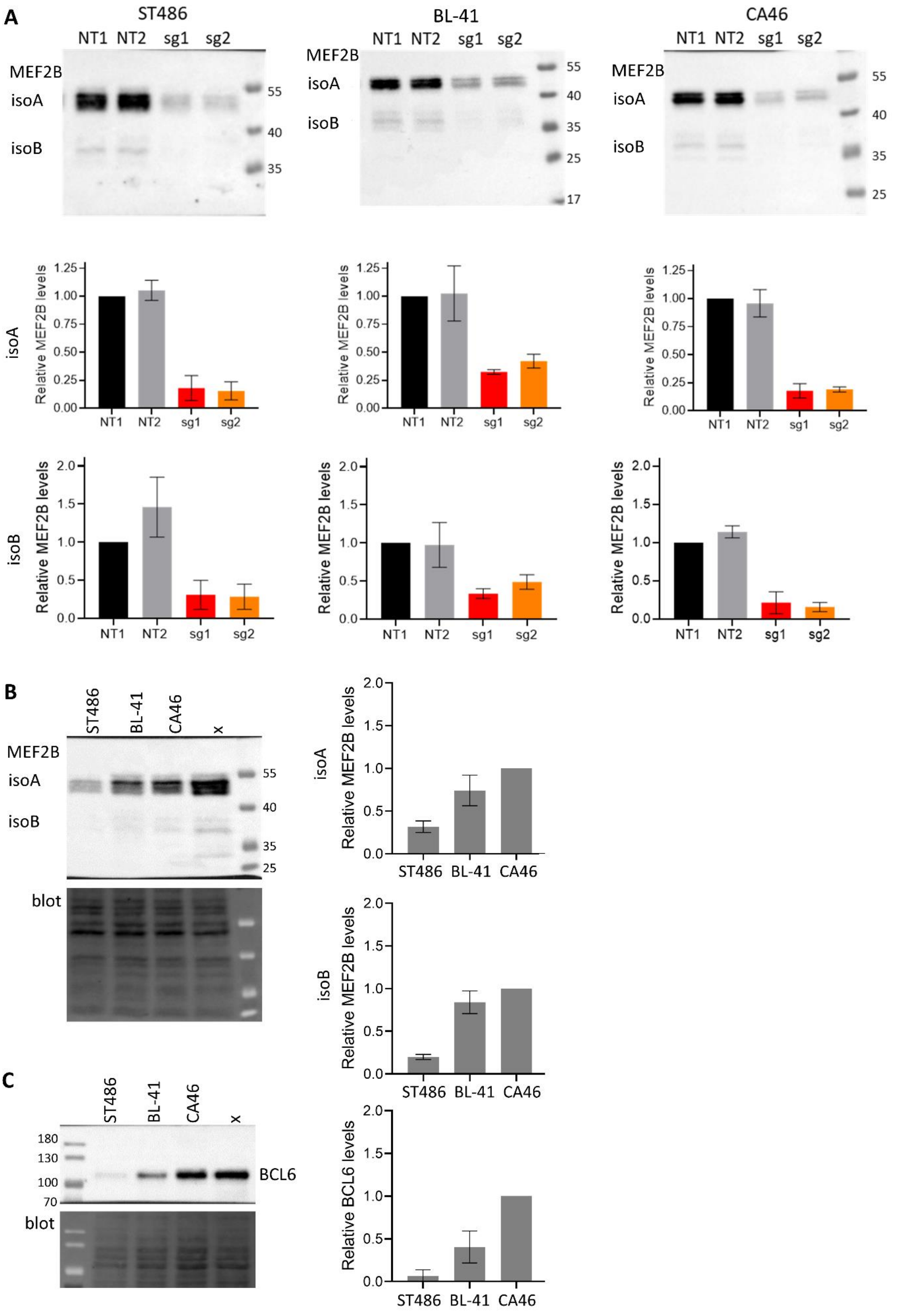
A
ST486
BL-41
CA46
NT1 NT2 sg1 sg2
MEF2B
isoA
isoB
55
40
35
25
17
Relative MEF2B levels
1.25
1.00
0.75
0.50
0.25
0.00
2.0
1.5
1.0
0.5
0.0
B
ST486 BL-41 CA46 x
blot
C
180
130
100
70
BCL6
Relative BCL6 levels

Supplementary Figure 2. Efficiency of MEF2B knockdown and endogenous MEF2B and BCL6 levels. (A) ST486, BL-41 and CA46 cells were transduced with lentiviral vectors carrying control (NT1: black, NT2: grey) or sgRNA sequences targeting MEF2B (sg1: red, sg2: orange). Western blots show downregulation of MEF2B levels upon MEF2B knockdown in the four BL cell lines. Quantification of the upper, stronger, and lower, weaker MEF2B bands was normalized to the total amount of protein loaded per lane, and the data were plotted relative to the NT1 control sample. A representative blot along with the mean ± SD of two independent experiments is shown, with each sample loaded twice on gel. Western blots show endogenous MEF2B (B) and BCL6 (C) levels in BL cell lines. Quantification was normalized to the total amount of protein loaded per lane, and the data were plotted relative to the levels in CA46. A representative blot along with the mean ± SD of two independent experiments is shown, with each sample loaded twice on gel. X – positive control.

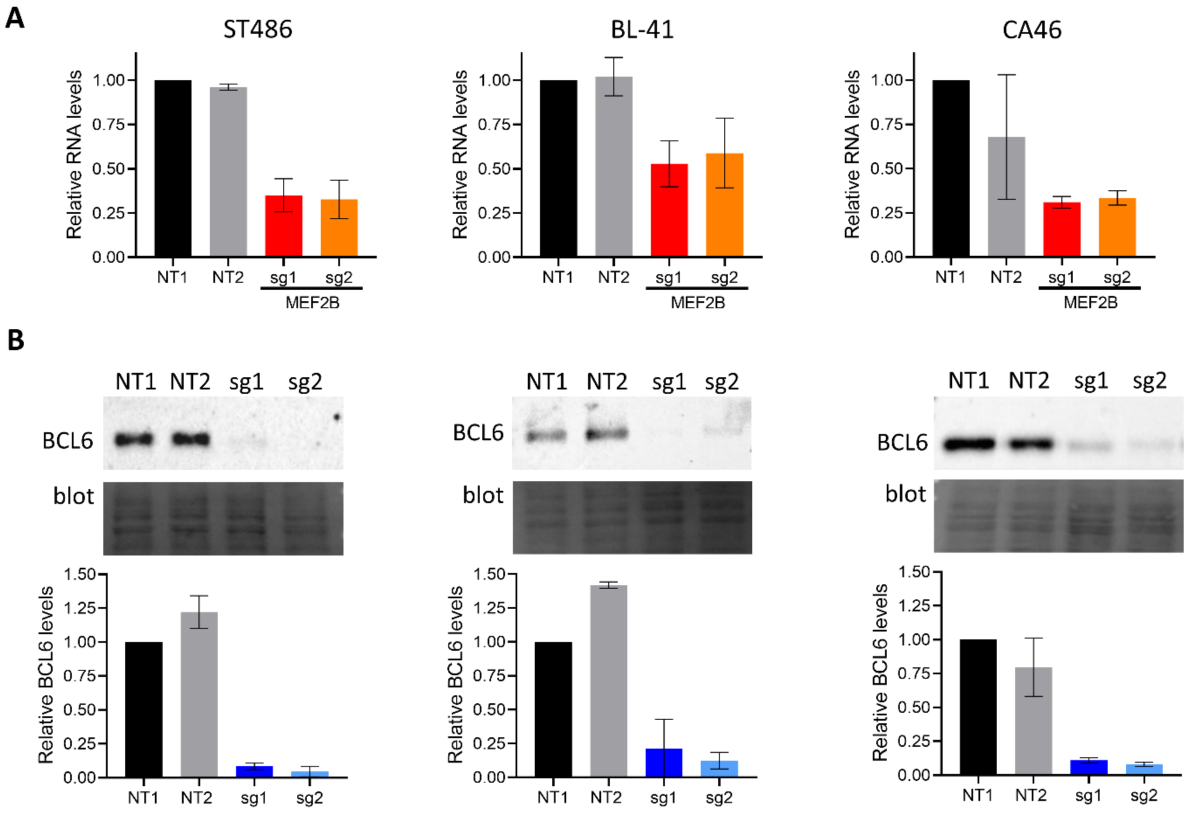

Supplementary Figure 3. BCL6 expression in BL cell lines upon MEF2B or BCL6 knockdown. (A) ST486, BL-41 and CA46 cells were transduced with lentiviral vectors carrying control (NT1: black, NT2: grey) or sgRNA sequences targeting MEF2B (sg1: red, sg2: orange). Relative BCL6 RNA levels upon knockdown of MEF2B were measured by qRT-PCR. Mean ± SD of two independent experiments is shown, and the data were normalized to the NT1 control. (B) ST486, BL-41 and CA46 cells were transduced with lentiviral vectors carrying control (NT1: black, NT2: grey) or sgRNA sequences targeting BCL6 (sg1: dark blue, sg2: light blue). Western blots show BCL6 protein levels upon BCL6 knockdown. Quantification was normalized to the total amount of protein loaded per lane, and the data were plotted relative to the NT1 control sample. A representative blot along with the mean ± SD of two independent experiments is shown, with each sample loaded twice on gel.

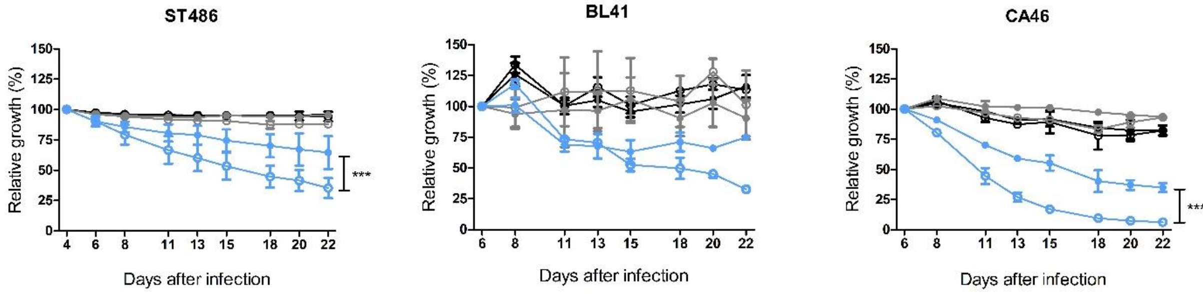

Supplementary Figure 4. Phenotype rescue of BCL6 knockdown upon BCL6 overexpression. ST486, BL-41 and CA46 cells were transduced with lentiviral vectors carrying control (NT1: black, NT2: grey) or sgRNA2 targeting BCL6 (blue) and cultured with 1 µg/ml (+D) or without doxycycyline (-D). Only sgRNA2 was used since it targets an exon-intron boundary which is not present in the overexpression vector. Relative growth of BL cells was measured by following the percentage of GFP+ cells over three weeks post-transduction, with the percentages normalized to day 4 or 6 after transduction. Mean ± SD of two independent experiments is shown. Significance was determined by mixed model analysis; *** p<0.001.

Supplementary Table 1: Sequences of shRNAs, sgRNAs and qRT-PCR primers

Name	Sequence (5'-3')
	shRNAs
NT1-S	GATCCGCTAAGGTCAAGTCGCCCCCGATCTTCCTGTCAGAGCGAGGGCGACTTAACCTTAGGG
NT1-AS	AATTCCCTAAGGTTAAGTCGCCCTCGCTCTGACAGGAAGATCGGGGGCGACTTGACCTTAGCG
NT2-S	GATCCCTAAGGTTAAGTCGCCCTCGCTCTAGCGAGGGCGACTTAACCTTAGGTTTTTG
NT2-AS	AATTCAAAAACCTAAGGTTAAGTCGCCCTCGCTAGAGCGAGGGCGACTTAACCTTAGG
ZDHHC11_sh2-S	GATCCGAGAAATGGGAGCACAGTATTCAAGAGATACTGTGCTCCCATTTCTCTTTTTG
ZDHHC11_sh2-AS	AATTCAAAAAGAGAAATGGGAGCACAGTATCTCTTGAATACTGTGCTCCCATTTCTCG
ZDHHC11_sh3-S	GATCCGGGAGCACAGTACTAGGAAATTTCAAGAGAATTTCCTAGTACTGTGCTCCCTTTTTG
ZDHHC11_sh3-AS	AATTCAAAAAGGGAGCACAGTACTAGGAAATTCTCTTGAAATTTCCTAGTACTGTGCTCCCG
	sgRNAs
NT1-S	CACCGACGGAGGCTAAGCGTCGCAA
NT1-AS	AAACTTGCGACGCTTAGCCTCCGTG
NT2-S	CACCGATCGTTTCCGCTTAACGGCG
NT2-AS	AAACCGCCGTTAAGCGGAAACGATG
MEF2B_sg1-S	CACCGAGGCCCCGTATACCACATCT
MEF2B_sg1-AS	AAACAGATGTGGTATACGGGGCCTC
MEF2B_sg2-S	CACCGCACCAACACTGACATCCTCG
MEF2B_sg2-AS	AAACCGAGGATGTCAGTGTTGGTGC
BCL6_sg1-S	CACCGCAAGACATCATGGCCTATCG
BCL6_sg1-AS	AAACCGATAGGCCATGATGTCTTGC
BCL6_sg2-S	CACCGTCCCTCACCTGCAGGCCATG
BCL6_sg2-AS	AAACCATGGCCTGCAGGTGAGGGAC
	qRT-PCR primers
ZDHHC11_all-F	CACTTGGGCTGCAACAAGAA
ZDHHC11_all-R	GGTGGGGTTTCAGGGTAGAAG
MEF2B-F	CAAAGGAATCGGCAGGTGAC
MEF2B-R	GGTTGGCGCTGTTGAAGATG
BCL6-F	CTGCAGATGGAGCATGTTGT
BCL6-R	TCTTCACGAGGAGGCTTGAT
TBP-F	GCCCGAAACGCCGAATAT
TBP-R	CCGTGGTTCGTGGCTCTCT
MVP-F	GGAGGTGCTGGAAAAGGACA
MVP-R	AAAGCCACTCATCTCCTGCC
CCL3-F	AGCCCGGTGTCATCTTCCTA
CCL3-R	TTCTGGACCCACTCCTCACT
CD82-F	AGAAAGCAGAACCCGCAGAG
CD82-R	GTCCTGGAGCTTCCTTCCAC
USP18-F	CCGTGGGAAACAGGTCTTGA
USP18-R	TCTGGGGGAAGTACAGGGAG
KLF2-F	CGGCAAGACCTACACCAAGA
KLF2-R	TGGTAGGGCTTCTCACCTGT
FAM107B-F	GCAGAAGTTGGAGCAGCTTG
FAM107B-R	TCACCTTCACAAACTCGGGG
CLDN16-F	TGCCTTTTTCTCTGCTGGGT
CLDN16-R	GGCATTTTGTGCTCACCTCC
GOLGA7B-F	GAGACTACAGCGATGGGACC
GOLGA7B-R	AGCCTCTGCGTAAAATCCGT
WKN2-F	TCTGTCCACCACGGTCATT
WKN2-R	GTCTAGGCGGGGTCAGTCA

S = sense sequence, AS = antisense sequence, F = forward primer, R = reverse primer. Restriction sites are underlined.

Supplementary Table 2: Antibodies used in Western blotting.

Name	Dilution	Catalog number	Company
anti-MEF2B, rabbit polyclonal antibody	1:500	HPA004734	Atlas antibodies
anti-BCL6 (D-8), mouse monoclonal antibody	1:200	sc7388	Santa Cruz Biotechnology
goat anti-rabbit immunoglobins/HRP	1:1000	P0448	Dako
rabbit anti-goat immunoglobins/HRP	1:1000	P0449	Dako
rabbit anti-mouse immunoglobins/HRP	1:1000	P0260	Dako

Supplementary Table 3: Probes up- or downregulated upon knockdown of all *ZDHHC11* transcripts. Probes were found to be differentially expressed upon knockdown of all *ZDHHC11* transcripts with FC ≥ 2.0. The order is the same as the hierarchical clustering shows in Fig. 1. FC and p-value of the microarray and a previously performed high-throughput screen with the human CRISPR Brunello knockout library performed in ST486 are presented.

Microarray					Brunello screen	
**ProbeName**	**GeneSymbol**	**FC**	**norm. p-value**		**FC**	**adj. p-value**
A_33_P3344204	ZDHHC11	-2.0	0.0017		1.2	0.1035
A_33_P3344405	MEF2B	-2.0	0.0009		-3.0	0.0000
A_33_P3314401	CLDN16	-2.2	0.0159		1.0	0.8499
A_33_P3351175	WNK2	-2.0	0.0033		1.1	0.4543
A_33_P3288942	FAM107B	-2.2	0.0227		1.4	0.0104
A_33_P3367361	FAM107B	-2.4	0.0035		1.4	0.0104
A_22_P00007182	GOLGA7B	-2.0	0.0019		-1.0	0.8594
A_32_P100830	KIF19	-2.6	0.0012		1.1	0.3844
A_21_P0013064	XLOC_l2_012899	-2.2	0.0134			
A_32_P54553	USP41	2.0	0.0025			
A_33_P3216898	LOC286367	2.2	0.0041			
A_23_P132159	USP18	2.1	0.0045		-1.0	0.9125
A_23_P1782	CD82	2.5	0.0151		1.2	0.1935
A_33_P3214343	PLCXD2	2.2	0.0041			
A_33_P3316273	CCL3	2.6	0.0003		1.1	0.3278
A_23_P74701	COL24A1	3.3	0.0004		1.2	0.3225
A_21_P0000451	SNORD116-27	2.2	0.0363			
A_21_P0005201	LOC101928211	2.3	0.0172			
A_33_P3355014	TMEM229B	2.5	0.0314		-1.4	0.0158
A_32_P220770	HCG26	2.0	0.0024			
A_23_P119196	KLF2	2.1	0.0029		1.6	0.0001
A_32_P33083	VCX2	2.1	0.0025		-1.4	0.0113
A_23_P13382	LSP1	2.3	0.0237		1.0	0.7807
A_33_P3243702	KLHL30	2.1	0.0002		-1.1	0.3619
A_23_P360744	RAG1	2.2	0.0003		-1.0	0.6732
A_21_P0000447	SNORD116-11	2.5	0.0015			
A_33_P3294608	MVP	3.2	0.0010		-1.2	0.2043
A_24_P244442	BSCL2	2.1	0.0033		-1.4	0.0236
A_21_P0000361	SNORA79	2.6	0.0486			
